# Resistance to the CCR5 Inhibitor 5P12-RANTES Requires a Difficult Evolution from CCR5 to CXCR4 Coreceptor Use

**DOI:** 10.1371/journal.pone.0022020

**Published:** 2011-07-08

**Authors:** Rebecca Nedellec, Mia Coetzer, Michael M. Lederman, Robin E. Offord, Oliver Hartley, Donald E. Mosier

**Affiliations:** 1 Department of Immunology and Microbial Science, The Scripps Research Institute, La Jolla, California, United States of America; 2 Department of Medicine, Case Western Reserve University, University Hospitals/Case Medical Center, Cleveland, Ohio, United States of America; 3 Mintaka Foundation for Medical Research, Geneva, Switzerland; 4 Department of Structural Biology and Bioinformatics, University of Geneva, Geneva, Switzerland; University of Cape Town, South Africa

## Abstract

Viral resistance to small molecule allosteric inhibitors of CCR5 is well documented, and involves either selection of preexisting CXCR4-using HIV-1 variants or envelope sequence evolution to use inhibitor-bound CCR5 for entry. Resistance to macromolecular CCR5 inhibitors has been more difficult to demonstrate, although selection of CXCR4-using variants might be expected. We have compared the in vitro selection of HIV-1 CC1/85 variants resistant to either the small molecule inhibitor maraviroc (MVC) or the macromolecular inhibitor 5P12-RANTES. High level resistance to MVC was conferred by the same envelope mutations as previously reported after 16–18 weeks of selection by increasing levels of MVC. The MVC-resistant mutants were fully sensitive to inhibition by 5P12-RANTES. By contrast, only transient and low level resistance to 5P12-RANTES was achieved in three sequential selection experiments, and each resulted in a subsequent collapse of virus replication. A fourth round of selection by 5P12-RANTES led, after 36 weeks, to a “resistant” variant that had switched from CCR5 to CXCR4 as a coreceptor. Envelope sequences diverged by 3.8% during selection of the 5P12-RANTES resistant, CXCR4-using variants, with unique and critical substitutions in the V3 region. A subset of viruses recovered from control cultures after 44 weeks of passage in the absence of inhibitors also evolved to use CXCR4, although with fewer and different envelope mutations. Control cultures contained both viruses that evolved to use CXCR4 by deleting four amino acids in V3, and others that maintained entry via CCR5. These results suggest that coreceptor switching may be the only route to resistance for compounds like 5P12-RANTES. This pathway requires more mutations and encounters more fitness obstacles than development of resistance to MVC, confirming the clinical observations that resistance to small molecule CCR5 inhibitors very rarely involves coreceptor switching.

## Introduction

Primary transmission of human immunodeficiency virus type 1 (HIV-1) infection is highly selective in two respects. First, it involves transmission of one or a few genetic variants in spite of the enormous genetic diversity of HIV-1 in the infected donor [1], [2], [3], [4]. Second, transmission of HIV-1 strains that use C-C chemokine receptor 5 (CCR5) as the entry coreceptor is highly favored [5], [6], [7], [8], [9], [10], consistent with the observation that individuals with deletion mutations in the coding region of CCR5 are highly resistant to HIV-1 infection [11], [12], [13]. These results imply that blocking HIV-1 binding to CCR5 is a viable strategy to prevent transmission, and non-human primate studies fully support this concept [14], [15], [16], [17], [18].

Two classes of CCR5 inhibitors have been developed. The first reported were amino terminal modifications of the CCR5 native ligand RANTES that caused CCR5 inhibition by internalization and sequestration [19], [20]. This class of macromolecular CCR5 inhibitors has continued to be developed to generate more potent inhibitors with more desirable characteristics [21], [22], [23]. The second class of CCR5 inhibitors comprise small molecules [24], [25], [26], [27], [28], most of which act by binding to a conserved site [29], [30], [31], [32] composed of multiple transmembrane domains of CCR5. The activity of the small molecule inhibitors is thought to be allosteric displacement of the extracellular domains of CCR5 so that the coreceptor binding regions of CD4-bound envelope no longer recognize the altered CCR5 configuration [33]. Maraviroc (Pfizer) was the first of these CCR5 inhibitors to be approved for clinical use, and has proven to be an effective antiviral agent in both treatment-naive and treatment-experienced individuals with predominately CCR5-using (R5) HIV-1 infection [34], [35], [36].

Resistance to small molecule CCR5 inhibitors arises by two distinct mechanisms. The most common is selection of pre-existing minor HIV-1 variants that can use CXCR4 for entry [37], [38], and therefore are not subject to inhibition. Selection of mutations that change the coreceptor use from CCR5 to CXCR4 has been demonstrated in vitro [39], but this mechanism of resistance is infrequent in patients treated with small molecule CCR5 inhibitors [40]. A less common resistance mechanism is mutation of the HIV-1 envelope to recognize the altered conformation of inhibitor-bound CCR5 [39], [41], [42], [43]. This mode of resistance usually results in cross-resistance to other small molecule CCR5 inhibitors [39], [41], [44], but not to the macromolecular CCR5 inhibitors [41] despite one early report to the contrary [45] that was later corrected [41]. Macromolecular CCR5 inhibitors can select for CXCR4-using viruses [46], [47], but no resistance to this class of inhibitors by HIV-1 that retains entry via CCR5 has been reported. The report of one chimeric SHIV162P3 variant with partial resistance to PSC-RANTES [48] has been disputed by a more recent study [49]. A high barrier to resistance would be advantageous in the development of anti-HIV-1 compounds targeting CCR5 for prevention to lower the risk associated with unrecognized HIV-1 infection. This prompted us to undertake in vitro selection experiments to compare development of resistance to the small molecule inhibitor maraviroc [39] and the macromolecular inhibitor 5P12-RANTES [23].

## Results

### 1. Selection of maraviroc-resistant HIV-1 CC1/85 variants

**Figure 1:**
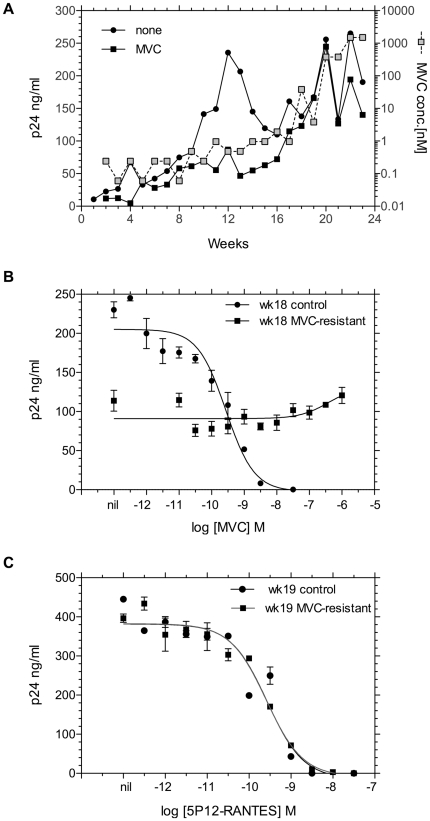
Development of resistance to maraviroc (MVC). **A.** Weekly increases in p24 capsid antigen (p 24 ng/ml, left y-axis) are plotted for control cultures with no CCR5 inhibitor (black circles) and cultures with increasing concentrations of MVC (black squares). The MVC concentration in nM (right y-axis) is indicated by grey squares. **B.** High level resistance to MVC after 18 weeks of selection. Data are mean p24 capsid antigen (± SE of triplicate cultures) plotted against increasing concentrations of MVC for HIV-1 CC1/85 from control cultures (filled circles) or MVC-selected cultures (filled squares). **C.** Resistance to MVC does not confer cross-resistance to 5P12-RANTES. Data presented as in panel B, but the inhibitor is 5P12-RANTES rather than MVC.

**Figure 2:**
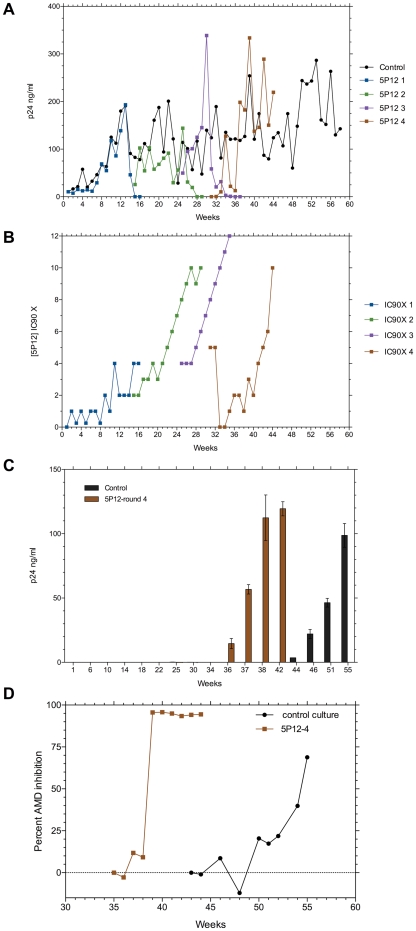
Development of resistance to 5P12-RANTES (5P12). **A.** Weekly increases in p24 capsid antigen during four successive rounds of selection: 5P12 1 (blue, weeks 1–15); 5P12 2 (green, weeks 13–25); 5P12 3 (purple, weeks 24–33); and 5P12 4 (brown, weeks 30–44). Control cultures with no inhibitor are shown in black filled circles. **B.** Increasing concentrations of 5P12 expressed as multiples of the 90% inhibitory concentration (IC_90_ = 0.12 nM) for each of the four rounds of selection, with colors matching panel A. **C.** Replication of viruses from indicated weeks of selection on activated CD4^+^ T cells from a CCR5Δ32 homozygous donor. Values are mean p24 capsid antigen levels (± SE of triplicate cultures) after 7 days of culture. **D.** Viruses from the indicated weeks of 5P12-RANTES round 4 of selection (5P12 4) or control cultures with no inhibitors were used to infect activated CD4^+^ T cells from normal donors in the presence of the CXCR4 blocking agent AMD3100 (AMD). The percent inhibition by AMD3100 of p24 capsid antigen levels after 7 days of culture is plotted versus the week of virus isolation.

We chose the HIV-1 R5 isolate CC1/85 [50] for these experiments because it has been used in multiple prior studies of resistance to small molecule CCR5 inhibitors [39], [41], [51], [52]. Virus was passaged weekly in fresh CD8 cell-depleted PBMC cultures containing increasing concentrations of either MVC or 5P12-RANTES. We used a very conservative dose escalation schedule because of multiple prior failures to generate HIV-1 isolates resistant to either PSC- or 5P12-RANTES (unpublished results). The concentration of MVC or 5P12-RANTES alternated between the IC_90_ concentration (0.24 nM for MVC; 0.12 nM for 5P12-RANTES) and the IC_50_ concentration (0.06 nM for MVC; 0.04 nM for 5P12-RANTES) for the first 8 weeks of selection (Figs. 1A and 2B). Subsequently the inhibitor concentration was gradually increased to 4× IC_90_ levels at week 15. During this period of slow increase in inhibitor concentration, cultures treated with MVC showed reduced virus replication compared to no inhibitor controls (Fig. 1A), whereas cultures treated with 5P12-RANTES showed virus replication comparable to no inhibitor controls between weeks 6 and 13 (Fig. 2A). Despite this indication of potential resistance to 5P12-RANTES by week 13, virus replication ceased during the following 2 weeks despite no increase in the concentration of 5P12-RANTES (Fig. 2A). During weeks 16 to 18 of selection, replication of virus in MVC-treated cultures began to approach control levels, and rapid escalation of MVC concentrations to as high as 1.5 µM failed to inhibit subsequent replication at weeks 18–23 (Fig. 1A).

We compared the sensitivity of HIV-1 CC1/85 cultured for 18 weeks in the absence of inhibitor to virus cultured for 18 weeks with escalating concentrations of MVC. The results (Fig. 1B) show that the infection of PBMC by control virus was fully sensitive to MVC inhibition, but the MVC-selected virus was resistant to the highest concentration of MVC tested. We thus confirm the results of Westby et al. [39] that a CC1/85 variant with greater than 1000-fold resistance to MVC can be selected in 16–18 weeks. We would not expect cross resistance between small molecule and macromolecular CCR5 inhibitors, but because of the past confusion in this area [45], we determined if the highly MVC-resistant CC1/85 variant had any change in sensitivity to 5P12-RANTES inhibition. The results of this experiment are shown in Fig. 1C. The MVC-resistant CC1/85 variant was fully sensitive to inhibition by 5P12-RANTES, but was cross-resistant to the allosteric CCR5 inhibitor TAK-779 (data not shown).

### 2. Sequence evolution associated with MVC-resistance or 5P12-RANTES-induced virus collapse

We isolated full length envelope (*env*) clones from the control, MVC-selected, or 5P12-RANTES-selected CC1/85 isolates to analyze sequence changes associated with resistance in the case of MVC or impending virus collapse in the case of 5P12-RANTES. We observed (Table 1) identical changes in the V3 sequences of both control virus and the highly MVC-resistant virus as reported by Westby et al. [39] in the majority of their *env* clones. This not only confirms their results but also suggests limited evolutionary pathways to MVC resistance. The sequences from the 5P12-RANTES-selected viruses showed a mixture of clones with V3 sequence changes identical to the control viruses and clones with additional V3 changes that may be deleterious in the absence of compensating mutations elsewhere in envelope [53]. Several *env* clones from week 13 of 5P12-RANTES selection with additional V3 mutations showed diminished ability to mediate infection of CCR5-expressing target cells in a single cycle assay (Table 1).

**Table 1:** V3 sequences of control, maraviroc-resistant, and 5P12-RANTES-selected CC1/85 HIV-1 viruses.

Virus	Selection	V3 sequence	CCR5 RLU^a^
CC1/85, start		CTRPNNNTRKSIHIGPGRAFYATGDIIGDIRQAHC	
	9 clones	-----------------------------------	7.678
CC1/85, week 18 control			
	5 clones	------Y------------L---------------	7.968
	3 clones	------Y------------W---------------	7.880
	1 clone	-------------------W---------------	4.945
CC1/85, week 18 MVC-resistant			
	3 clones	------------------T------V---------	8.132
	2 clones	------------------S------V---------	8.083
	3 clones	------------------S----------------	8.512
	1 clone	------------------T----------------	8.209
[MVC-resistant clones; Westby *et al.*]			
	6 clones	------------------T------V---------	
	1 clone	------------------S------V---------	
CC1/85, week 13 control			
	4 clones	------Y------------L---------------	7.969
	3 clones	-------------------L---------------	7.752
CC1/85, week 13 5P12-RANTES			
	8 clones	------Y------------L---------------	7.910
	1 clone	-----------------------------------	7.950
	1 clone	------------L--------P-------------	4.713
	1 clone	------Y------------L-------E-------	4.241
	1 clone	------Y-K-----E--K-L-------EN-K----	4.395

a Log_10_ relative light units (RLU) in single cycle infection of NP-2.CD4.CCR5 cells mediated by envelope (*env*) clones with the indicated V3 sequence. Mean values for multiple *env* clones with the same V3 sequence, representative single values for individual clones. Note that *env* clones with the same V3 sequence may differ in sequence in other regions of envelope.

### 3. Prolonged selection attempts to generate 5P12-RANTES-resistant CC1/85 variants

An effort to rescue 5P12-RANTES resistant virus from week 13, the last time point with robust virus replication, was made (indicated by 5P12-RANTES 2 in Fig. 2A). That virus was propagated without inhibitor for 1 week, and then 5P12-RANTES selection restarted at 2× IC_90_, the concentration originally present at week 13. Virus replication continued at reduced levels compared to no inhibitor controls until week 25, when raising the 5P12-RANTES concentration to 8× IC_90_ led to a second collapse of replication (Fig. 2A). We restarted 5P12-RANTES selection a third time using virus from week 24 that survived 4× IC_90_ concentration, and slowly escalated 5P12-RANTES levels from weeks 25 to 33 of passage (Fig. 2B). Virus replication increased until week 30, and then diminished when 8× IC_90_ concentrations were exceeded (Fig. 2A). A fourth (and final) round of selection was started with virus from week 30, which was initially cultured with 5× IC_90_ concentrations of 5P12-RANTES. Virus replication was almost completely inhibited by these concentrations, so no inhibitor was added for 2 subsequent weeks, and then a more conservative dose escalation restarted (Fig. 2B). A major increase in virus replication occurred between weeks 34 and 36 of the fourth round of selection (Fig. 2A). At this time, an increase in cytopathic effect was noted, suggesting a possible switch to CXCR4 use. This was evaluated by using sequential virus isolates from weeks 30 to 44 of selection by 5P12-RANTES and weeks 36–55 from control cultures to infect activated CD4 T cells from a CCR5 Δ32 homozygous donor. Fig. 2C shows that CCR5-independent infection by 5P12-RANTES selected CC1/85 virus was first detected at week 36 of round 4 of selection, and improved substantially during the ensuing six weeks. Surprisingly, a similar but delayed evolution of CCR5-independent infection occurred in virus from control cultures without any CCR5 inhibitor added (Fig. 2C) between weeks 44 and 55 of culture. We confirmed that infection was mediated by the CXCR4 coreceptor by inhibiting infection with the CXCR4-specific agent AMD3100 (Fig. 2D). AMD3100 completely blocked infection by HIV-1 CC1/85 from weeks 39 and later of 5P12-RANTES selection. By contrast, AMD3100 was only partially inhibitory for virus recovered from control cultures, suggesting a mixture of CCR5- and CXCR4-using viruses. These data indicate that resistance to 5P12-RANTES was generated by the selection of virus variants using CXCR4 for entry, but that this occurred in the context of a virus population that was evolving towards CXCR4 use even in the absence of added CCR5 inhibitors.

### 4. Sequence correlates of CXCR4 use

**Figure 3:**
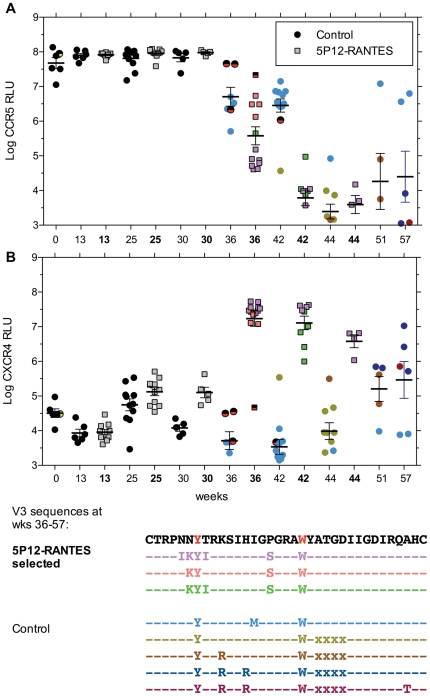
Entry efficiency via CCR5 versus CXCR4. Full length envelope (*env*) clones were generated after the indicated weeks of selection by 5P12-RANTES (grey or colored filled symbols, weeks in bold) or control cultures with no inhibitors (solid symbols, weeks not bold), and used to mediate entry of an *env*-negative reporter construct into NP-2.CD4.CCR5 target cells (square symbols, panel **A**) or NP-2.CD4.CXCR4 target cells (circles, panel **B**). Entry efficiency was determined by luciferase expression, which is plotted as log_10_ relative light units (log RLU). Each symbol is the entry result for an individual *env* clone, and the mean ± SE for all *env* clones is indicated by the bold horizontal line and error bars. Rapid sequence evolution occurred between weeks 30 and 36 of culture, and V3 sequences that evolved are color-coded in the legend, and matched to the entry data for each *env* clone with the designated sequence. The top V3 sequence predominated in both the control and 5P12-RANTES selected viruses isolated at week 30, but only three *env* clones retained that sequence at week 36 (indicated by black/red symbols). This V3 sequence differed from the starting CC1/85 sequence by the two amino acids indicated in red. The four amino acid deletion in V3 observed in control cultures is indicated by xxxx. The starting sequence that gave rise to CXCR4-using variants (clone #11) is indicated in panel A and B at week 0 by the half-filled symbol (see Fig. 4). The single clones from week 13 with poor entry function on both CCR5 (Table 1) and CXCR4 are not shown in this figure.

We explored the evolution of CCR5 and CXCR4 use following 5P12-RANTES selection by evaluating single cycle infection mediated by CC1/85 *env* clones isolated after 13 (round 1), 25 (round 2), 30 (round 3), and 36–44 (round 4) weeks of virus replication (Fig. 3). Entry via CCR5 remained robust with no difference between control and 5P12-RANTES selected *env* clones until week 36 (Fig. 3A). By contrast, entry mediated by CXCR4 was significantly improved for most *env* clones isolated after 25 (p = 0.0016) or 30 weeks (p = 0.0231) of 5P12-RANTES selection compared to entry mediated by CC1/85 *env* clones from week 0 (Fig. 3B). However, some *env* clones from control cultures also showed improved entry via CXCR4 during this time period. At week 36, there were marked changes in entry efficiency that were associated with the emergence of distinctive V3 sequences (legend to Fig. 3). First, the majority of *env* clones from round 4 of 5P12-RANTES selection showed reduced entry via CCR5 (Fig. 3A) and much improved entry via CXCR4 (Fig. 3B). *Env* clones from control cultures at week 36 also showed a more modest reduction in entry via CCR5, but no improvement in entry via CXCR4, and most had a unique V3 sequence that persisted from week 36 to week 57. At week 42 of 5P12-RANTES selection, CCR5 entry had further declined while robust entry via CXCR4 persisted, and one of two V3 sequences was replaced by a closely related variant. *Env* clones from week 42 of control cultures showed a single example of improved CXCR4 entry and decreased CCR5 entry that was associated with a unique V3 sequence with a four amino acid deletion (loss of ATGD, positions 318–321). *Env* clones from week 44 of 5P12-RANTES selection had identical V3 sequences associated with very poor entry via CCR5 and robust entry via CXCR4. In addition to the six amino acid changes in the V3 region, these *env* clones also had two shared substitutions in C2, I272V and N277D. The latter substitution eliminates a potential N-linked glycosylation site. Control *env* clones from week 44 were mainly derived from the four amino acid deletion variant first identified at week 42, and were still relatively poor at entry via CXCR4. These were replaced with closely related sequence variants that added charged arginine residues in V3 that improved their entry function via CXCR4. However, some sequence variants first identified at week 36 persisted, and these were better at entry via CCR5 than CXCR4. These results are consistent with the AMD3100 inhibition results in Fig. 2D, confirming the co-existence of both R5 and X4 variants within the control virus population.

### 5. Envelope sequence evolution to CXCR4 use

**Figure 4:**
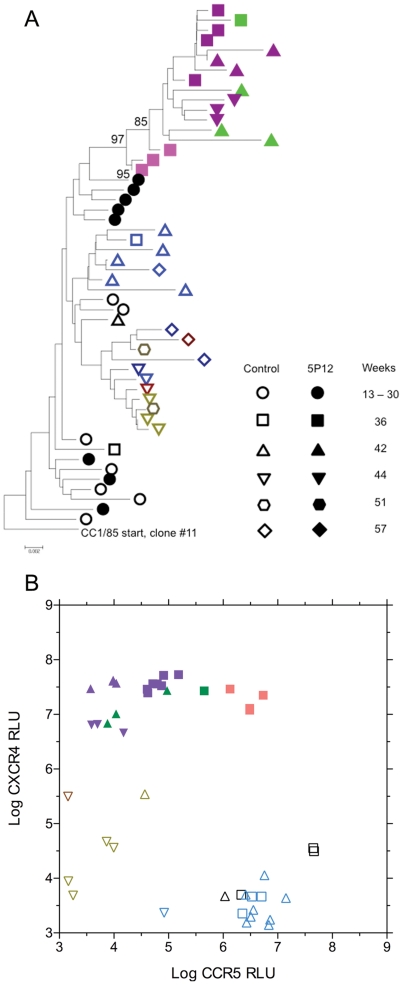
Env (gp160) sequence evolution to CXCR4 use. **A.** A phylogenetic tree representing the *env* clones that evolved from CCR5 to CXCR4 use. The tree is rooted with one of two variants found in the starting CC1/85 virus population with a TNNTxN motif sequence at position 459–465 (HXB2 numbering) in C5 instead of NDTSGT. All *env* clones that developed CXCR4 use were derived from this founder sequence. The weeks at which the *env* clones were isolated is indicated by the symbol legend, and the V3 sequence is indicated by the color given in the legend to Fig. 3. All *env* clones from week 36 and later of 5P12-RANTES selection were capable of using CXCR4 (see below), whereas only a subset of control *env* clones from week 44 or later were capable of entry via CXCR4. **B.** Entry data for *env* clones from weeks 36, 42, and 44 either from control cultures (open symbols) or 5P12-RANTES containing cultures (closed symbols, weeks depicted as in Fig. 4A). The symbols are color coded as in Figs. 3 and 4A.

Since both prolonged selection with 5P12-RANTES and prolonged culture without added CCR5 inhibitors led to the emergence of CXCR4-using viruses, we were interested in determining if the evolution of CXCR4 use was similar or different for the two groups. Full length gp160 sequences were obtained for the majority of *env* clones used in single cycle infection assays depicted in Fig. 3. The phylogenetic tree of the amino acid sequence of all *env* clones on the pathway to CXCR4 use in both 5P12-RANTES selected viruses and control viruses is shown in Fig. 4A. The tree is rooted by one of two distinct *env* sequences present in the starting CC1/85 isolate with a motif sequence in the C5 region that was retained by all later *env* clones, both CCR5- and CXCR4-using, on this tree. The sequences from viruses under 5P12-RANTES selection cluster together at the top of the tree, and are closely related to a set of CCR5-using *env* clones present at weeks 25 and 30 of selection. There was a mean divergence of 3.8% from the starting sequence at week 36 of selection. The sequences of control viruses capable of CXCR4 use clustered in the middle of the tree, and had a mean divergence of 2.8% from the starting sequence at week 44 of culture. The *env* clones that retained only CCR5 use clustered together closer to the starting sequence. Fig. 4B gives the entry efficiency via CCR5 and CXCR4 mediated by *env* clones from weeks 36, 42, and 44 for both the 5P12-RANTES selection and control viruses. The data plotted in Fig. 4B emphasizes that the sequence differences depicted in Fig. 4A led to distinct entry phenotypes. All *env* clones from cultures under 5P12-RANTES selection (except one from week 36, depicted by the red/black filled symbol in Fig. 3) mediated robust entry via CXCR4 and diminishing entry via CCR5. By contrast, *env* clones from control cultures were better at mediating entry via CCR5 and entry via CXCR4 improved only modestly as entry via CCR5 declined. The selection pressure exerted by 5P12-RANTES thus led to a transient appearance of R5X4 viruses, followed by their evolution to X4 only. The virus population in control cultures at the same time points was more complex, with a predominance of R5 viruses, some R5X4 viruses, and only a few weak X4 viruses.

The sequence evolution from CCR5 to CXCR4 coreceptor use was driven by mutation, and the rate of mutation is proportional to the number of viral replication cycles, not the weeks of virus culture. Replication was initially reduced by addition of MVC (see Fig. 1A) or 5P12-RANTES (see Fig. 2A) in each round of selection. Table 2 presents the translation of cumulative p24 capsid antigen levels (AUC, area under curve from Fig. 2A) to cycles of replication to correct for the reduced replication in the presence of inhibitors. These corrections indicate that the emergence of CXCR4 use at week 36 during round 4 of 5P12-RANTES selection occurred after 150 cycles of replication, whereas the spontaneous emergence of CXCR4 use in control cultures at week 44 occurred after 308 cycles of replication (Table 2). We also calculated the observed versus expected number of nucleotide substitutions for the envelope gene using the mutation rate/base pair/cycle recently determined by Abram et al. [54]. The result (Table 2) suggests strong directional selection for the mutations associated with resistance to 5P12-RANTES, whereas the evolution to CXCR4 use in control cultures is more consistent with neutral drift. We also determined if the levels of RANTES present in the medium during virus passage might select for CXCR4 use, and the peak values were too low (<1.15 ng/ml) in these CD8 T cell-depleted cultures to inhibit virus entry via CCR5 [55].

**Table 2:** Calculation of virus replication cycles during selection.

	Control	5P12 round 1	5P12 round 2	5P12 round 3	5P12 round 4
**First week**	1	1	15	25	31
**Last week**	58	15	28	36	44
**AUC** 1	7044	802	851	945	1720
**Increase/week**	121.45	53.46	65.46	85.91	132.21
**% Control**	(100)	44.02	53.90	70.74	108.94
**Replication Cycles** 2	406	46.22	49.05	54.47	99.14
**Weeks to X4 switch**	44				36
**Cycles to X4 switch**	308				150
**Expected mutations** 3	11.01				5.36
**Observed mutations** 4	13.67				23.88

1 AUC; area under curve for cumulative increase in capsid p24 antigen shown in Fig. 2A for the indicated number of weeks (first to last).

2 Calculation of replication cycles assume 1 cycle/day for HIV-1 CC1/85 in control cultures [66], and is corrected for weeks of replication (i.e., allowing for pauses) and diminished p24 levels (% control) for cultures under 5P12-RANTES (5P12) selection.

3 Expected mutations are calculated based on the rate found by Abram *et al.* [54] of 1.4×10^−5^ mutations/bp/cycle and the 2553 bp target envelope gene.

4 Observed mutations are the mean number of nucleotide mutations observed in all envelope molecular clones with confirmed entry via CXCR4.

## Discussion

Our primary finding is that resistance to the small molecule CCR5 inhibitor MVC could be generated in the same time frame and by the same apparent mechanism as previously described [39], whereas resistance to the macromolecular CCR5 inhibitor 5P12-RANTES developed only after four successive rounds of selection (the first three resulting in virus extinction) by coreceptor switching to CXCR4. However, this route to resistance must be viewed in the context of control cultures where virus evolution to the use of CXCR4 as a coreceptor also occurred, albeit at a slower pace, with fewer and different envelope mutations, and with better preservation of CCR5 use.

The MVC-resistance results presented in Figure 1 confirm the prior observations [39], [45], [51] that HIV-1 CC1/85 develops resistance to small molecule CCR5 inhibitors while retaining use of CCR5. Other HIV-1 isolates, notably HIV-1 SF162, can escape small molecule CCR5 inhibitors by either switching to CXCR4 [39] or by selection of pre-existing CXCR4-using variants [56]. In vitro selection of small molecule CCR5 inhibitor-resistant isolates that retain use of drug-bound CCR5 is generally associated with relatively few mutations in the V3 region [39], [57], although there are notable exceptions to this finding [58]. Our replication of the results of Westby et al. [39] confirms that only two replacement mutations in V3 contribute substantial resistance to MVC (Table 1). We did not observe the three additional changes noted by Westby et al. (T163K, N355Y, S405A) in MVC-resistant *env* clones, nor did we isolate the minor population of MVC-resistant viruses with three mutations in V3, but we did observe the N355Y and S405A changes in a subset of *env* clones from control cultures. We postulate that the two V3 mutations necessary for MVC resistance of the CC1/85 isolate are fewer than the eight critical mutations (six in V3 and two in C2) associated with resistance to 5P12-RANTES by CXCR4 use (Figs. 3 and 4), and thus that recognition of MVC-bound CCR5 is favored over coreceptor switching in the development of resistance in vitro.

Extensive sequence evolution in the V3 region and compensatory changes in other regions of *env* are generally required for the CCR5 to CXCR4 coreceptor switch [53], [59], [60], and was observed in these experiments both with the selective pressure of adding 5P12-RANTES as well as in control virus cultures. Acquisition of resistance to AOP-RANTES was previously observed to be associated with coreceptor switching that required only two V3 mutations [46], but that result was confounded by the choice of the virus isolate which was derived from a CXCR4-using parental strain that was converted to CCR5 use by V3 mutation, and the CXCR4-using variant quickly reverted to CCR5 use when AOP-RANTES was removed [61]. By contrast, 5P12-RANTES selected, CXCR4-using variants in the current experiments failed to revert to CCR5 use when propagated for up to two months in the absence of inhibitor. We chose to study resistance to 5P12-RANTES in the CC1/85 strain because it was a patient isolate previously used in multiple studies of resistance to CCR5 inhibitors [39], [41], [51], [52] and it displayed robust replication in vitro (see Fig. 2A). However, virus isolated from the same subject a year later (CC2/86) had undergone coreceptor switching to CXCR4 [50], so the CC1/85 population of viruses may have contained some intermediates that were exploring CXCR4 entry. The data in Fig. 3B indicate that many of the *env* clones from the starting CC1/85 virus stock could mediate entry via CXCR4 at levels well above background, even though no virus capable of infecting primary T cells from a CCR5 Δ32 homozygous donor was isolated until week 36 of 5P12-RANTES selection or week 44 from control cultures (Fig. 2C). The fact that CXCR4 use evolved in control cultures by 44 weeks of passage (as compared to 56 weeks in the infected subject) is consistent with precursors with modest CXCR4 use being present in the starting CC1/85 isolate. Prolonged culture of virus is also known to influence the entry phenotype [62], and it is clear from Fig. 4A that virus from control cultures underwent considerable sequence divergence although not as much as virus under 5P12-RANTES selection pressure. Virus under selection by 5P12-RANTES showed strong evidence of positive selection of envelope mutations, whereas virus isolated from control cultures had fewer mutations and did not appear to be under strong selection (Table 2). Indeed, the agreement between the predicted number of nucleotide substitutions derived from an artificial lacZ target sequence [54] and the number of observed mutations in the *env* gene of control virus (Table 2) is an independent confirmation of the in vitro HIV-1 mutation rate. The combination of V3 and C2 mutations associated with coreceptor switching in both the 5P12-RANTES selected variants and the X4 variants from control cultures have not been observed previously, although the deletion of the ATGD V3 sequence that appeared in control virus is similar to a laboratory deletion of IIGD (Δ26–29) in HIV-1 clone R3A that was also observed to impair entry via CCR5 but preserve entry via CXCR4 [63].

A striking result of our studies was the repeated collapse of virus replication after the development of apparent partial resistance to 5P12-RANTES. Some *env* clones from weeks 13 of 5P12-RANTES selection showed diminished entry function via CCR5 (Table 1) as did many *env* clones from week 36 and later (Fig. 3A). A minority of *env* clones from control (no inhibitor) cultures also mediated poor entry via CCR5 (Table 1). These results suggest that selection by 5P12-RANTES may result in some viral variants with reduced binding of CCR5, which would result in increased sensitivity to inhibition, a plausible explanation for their transient appearance. It is worth noting that we have previously observed increased sensitivity to CCR5 inhibitors at the time of coreceptor switching in an infected subject [60], and many coreceptor switch intermediates show diminished entry via both CCR5 and CXCR4 [59]. This observation is confirmed by the data shown in Fig. 3, where it is obvious that entry via CCR5 begins to diminish as CXCR4 entry is gained in both 5P12-RANTES selected and control cultures. Thus one explanation for the virus extinction at the end of the first three rounds of selection is that evolution towards CXCR4 use involves intermediates with increased sensitivity to CCR5 inhibitors.

These results confirm that the only apparent route to resistance to macromolecules like 5P12-RANTES appears to be virus evolution to CXCR4-mediated entry. In the setting of prevention trials, the blocking of CCR5 should prevent infection and the initiation of the long and difficult evolution to CXCR4 use. Neither small or large molecule CCR5 inhibitors would prevent the rare transmission of X4 variants [6], [9].

## Materials and Methods

### Ethics statement

Whole venous blood was collected from anonymous donors participating in The Scripps Research Institute volunteer donor pool. Written informed consent was obtained from all donors and/or their legal guardians, and the protocol was approved by the Scripps Health Institutional Review Board.

### CCR5 inhibitors and virus

Maraviroc (MVC) was kindly provided by Hernan Valdez (Pfizer, New York, NY). 5P12-RANTES was prepared by chemical synthesis as described [23]. The R5 HIV-1 isolate CC1/85 was kindly provided by Shawn Kuhmann and John Moore (Cornell University, New York, NY).

### Virus passage

Virus was propagated using pooled CD8^+^ T cell-depleted PBMC from 4 donors who were heterozygous for the CCR5 Δ32 mutation to increase the susceptibility of target cells to CCR5 inhibitors. The same 4 donors were used throughout the experiment. PBMC were CD8^+^T cell-depleted by negative selection using anti-CD8 antibody (BD, Palo Alto, CA) and binding to BioMag beads (Qiagen), followed by stimulation for two days with 2 µg/ml PHA followed by two days with 20 units/ml IL-2 in RPMI 1640 medium with 10% fetal bovine serum (FBS). Fifty percent inhibitory concentrations (IC_50_) and 90 percent inhibitory concentrations (IC_90_) of MVC or 5P12-RANTES were determined using the pooled CD8^+^ T cell-depleted PBMC before starting the experiment. Infection was started by adding 1000 TCID_50_/ml CC1/85 to 20 ml of PBMC at 2×10^6^/ml. Every week 5 ml of cell-free supernatant from the prior week's culture was added to 15 ml of freshly stimulated CD8-depleted PBMC [51] that were untreated (control) or had been incubated with MVC or 5P12-RANTES at the indicated concentration for 30 minutes. Five ml of IL-2-containing medium was added midweek to support robust virus replication. Each week medium and cells were frozen for subsequent assays, and p24 viral capsid antigen was measured by ELISA (Perkin Elmer, Waltham, MA).

Blood from a CCR5 Δ32 homozygote was obtained from an anonymous adult donor participating in the volunteer donor program of The Scripps Research Institute. PBMC were separated by Ficoll-Hypaque density sedimentation and stimulated two days with 2 µg/ml PHA followed by 2 days with 20 units/ml IL-2 in RPMI 1640 medium with 10% FBS. These cells were used to check infectivity of viruses from the control and the 5P12-RANTES treated cultures.

### Envelope Cloning and Pseudovirus Coreceptor Typing

The gp160 envelope gene was amplified from cellular DNA using primer pair envA and envM as previously described [64]. The 3KB PCR fragments were cloned into an expression vector (pcDNA3.1, Invitrogen) and co-expressed with the NL4.3 *env*-negative, luciferase-positive reporter plasmid [65] in 293T cells. Coreceptor use of viruses or envelope clones was evaluated by infection of NP2.CD4.CCR5 and U87.CD4.CXCR4 cell lines that were maintained in DMEM with 10% FBS, 1 µg/ml puromycin and 500 µg/ml of G418. The luciferase activities were determined as previously described [59], and are reported as relative light units (RLU).

### Genotypic analysis

Sequences were compiled and visualized using Lasergene 8.1 software (DNASTAR, Madison, WI). Sequences were aligned with ClustalX and manually edited using BioEdit (version 7). Phylogenetic analysis were determined using MEGA (version 3.1; Molecular Evolutionary Genetics Analysis). Sequences of full length *env* clones will be deposited to GenBank upon acceptance of the manuscript.
